# Burden of Comorbidities in Patients with OSAS and COPD-OSAS Overlap Syndrome

**DOI:** 10.3390/medicina57111201

**Published:** 2021-11-04

**Authors:** Athanasios Voulgaris, Kostas Archontogeorgis, Athanasia Pataka, Alexandros N. Flaris, Paschalis Ntolios, Maria R. Bonsignore, Sophia Schiza, Paschalis Steiropoulos

**Affiliations:** 1 MSc Program in Sleep Medicine, Medical School, Democritus University of Thrace, 68100 Alexandroupolis, Greece; k.archontogeorgis@yahoo.it (K.A.); steiropoulos@yahoo.com (P.S.); 2 Department of Pneumonology, Medical School, Democritus University of Thrace, 68100 Alexandroupolis, Greece; pascnt@hotmail.com; 3 Respiratory Failure Unit, George Papanikolaou General Hospital, Aristotle University, 57010 Thessaloniki, Greece; patakath@yahoo.gr; 4 Department of Surgery, Tulane University, School of Medicine, New Orleans, LA 70112, USA; alexander.flaris@gmail.com; 5 Institute of Biomedicine and Molecular Immunology, National Research Council (CNR), 90146 Palermo, Italy; mariarosaria.bonsignore@unipa.it; 6 Biomedical Department of Internal and Specialistic Medicine (DIBIMIS), University of Palermo, 90133 Palermo, Italy; 7 Sleep Disorders Unit, Department of Respiratory Medicine, Medical School, University of Crete, 71500 Heraklion, Greece; schiza@med.uoc.gr

**Keywords:** obstructive sleep apnea syndrome, chronic obstructive pulmonary disease, overlap syndrome, comorbidities, cardiovascular disease

## Abstract

*Background and Objectives*: Obstructive sleep apnea syndrome (OSAS) and chronic obstructive pulmonary disease (COPD) are usually associated with multi-morbidity. The aim of this study was to retrospectively investigate the prevalence of comorbidities in a cohort of patients with OSAS and COPD-OSAS overlap syndrome (OS) patients and to explore differences between these two groups. *Materials and Methods*: Included were consecutive OS patients and OSAS patients who had been referred to our sleep laboratory, and were matched in terms of sex, age, BMI, and smoking history. Presence of comorbidities was recorded based on their medical history and after clinical and laboratory examination. *Results*: The two groups, OS patients (*n* = 163, AHI > 5/h and FEV_1_/FVC < 0.7) and OSAS patients (*n* = 163, AHI > 5/h, and FEV_1_/FVC > 0.7), did not differ in terms of apnea hypopnea index (*p* = 0.346), and oxygen desaturation index (*p* = 0.668). Compared to OSAS patients, OS patients had lower average SpO_2_ (*p* = 0.008) and higher sleep time with oxygen saturation <90% (*p* = 0.002) during sleep, and lower PaO_2_ (*p* < 0.001) and higher PaCO_2_ (*p* = 0.04) in wakefulness. Arterial hypertension was the most prevalent comorbidity for both OS and OSAS, followed by dyslipidemia, cardiovascular disease (CVD) and diabetes. OS was characterized by a higher prevalence of total comorbidities (median (IQR):2 (1–3) vs. 2 (1–2), *p* = 0.033), which was due to the higher prevalence of CVD (*p* = 0.016) than OSAS. No differences were observed in other comorbidities. *Conclusions*: In OS patients, nocturnal hypoxia and impaired gas exchange in wakefulness are more overt, while a higher burden of CVD is observed among them in comparison to sex-, age- and BMI-matched OSAS patients.

## 1. Introduction

Obstructive sleep apnea (OSA) and chronic obstructive pulmonary disease (COPD) are highly prevalent in the general population, with an occurrence of approximately 10% for each [1]. Nevertheless, both are frequently under-diagnosed, leading to several medical complications and increased social and financial burden [2,3]. OSA is defined as complete (apneas) or partial (hypopneas) airflow cessation during sleep leading to nocturnal oxygen desaturations, sleep fragmentation, and daytime sleepiness [4], while COPD is characterized by persistent airflow obstruction in association with a history of risk factors: mainly smoking as well as environment pollution and occupational exposure to dusts and fumes [5].

Coexistence of obstructive sleep apnea syndrome (OSAS) and COPD in the same patient was first introduced by Flenley in 1985 and is termed as overlap syndrome (OS) since then [6]. The prevalence of OS is estimated at 1–3.6% in the general population, with a higher occurrence among those patients who exhibit either OSAS or COPD [7]. Patients with OS demonstrate poorer quality of life than age-matched COPD patients and are more likely to express worse indices of sleep hypoxia than OSAS patients [7]. Furthermore, OS patients are exposed to higher risk for death when OSAS is left untreated as compared with COPD only individuals [8], while increased healthcare utilization is more often observed in OS than COPD patients [9]. 

Both diseases share and/or interact with common risk factors such as increasing age, male gender, smoking, and obesity [10]. Interestingly, accumulating evidence also indicates that both OSAS and COPD are usually accompanied by several comorbidities; in particular advanced age and the aforementioned shared risk factors are the main cause for this [11,12]. In addition, recent studies have shown that patients with OS are also frequently affected from cardiometabolic disorders [13,14]. Specifically, OS is often related with either an increased risk of cardiovascular disease (CVD) [15], or with established CVD [16], and mainly pulmonary hypertension (PH) [17], which is the best studied comorbidity in this specific population. Decreased oxyhemoglobin saturation during sleep and wakefulness, impaired lung function, increased oxidative stress, as well as tobacco exposure, reduced physical activity and increased body mass index (BMI) are amongst the recognized factors which contribute to the development of CVD in patients with OS [10]. In any case, should patients be afflicted by both OSAS and COPD, the result is expected to be more detrimental in terms of coexisting diseases than patients who suffer from either OSAS or COPD alone. 

However, until now there has been a lack of evidence about the exact prevalence of comorbidities, apart from CVD, in patients with OS. The aim of this case–control study was to assess the prevalence of comorbidities in a group of consecutive patients diagnosed with OS, at the time of their first evaluation at a tertiary university hospital, compared to patients with OSAS, and to explore potential differences between these two groups.

## 2. Materials and Methods

### 2.1. Patients

A total of 163 consecutive patients who were diagnosed with OS from 2011 to 2018, at the sleep laboratory of the University General Hospital of Alexandroupolis, Greece, were retrospectively enrolled in the study. The following inclusion criteria were applied: consecutive patients with COPD and OSAS, i.e., OS, newly diagnosed via pulmonary function testing and polysomnography, aged 40 years or older; current or ex-smokers with at least ten pack-years smoking history; and available and complete medical records for assessment of comorbidities. The exclusion criteria were central sleep apnea syndromes and inability to extract information on patients’ comorbid illnesses. 

After applying the abovementioned criteria which resulted in the enrollment of 163 consecutive OS patients, a one-to-one sex-, age- and BMI- matching procedure resulted in another 163 patients, diagnosed with OSAS without COPD in the same period. The study protocol was approved by the institutional ethics committee and all procedures were conducted in accordance with the Helsinki Declaration of Human Rights [18]. Written informed consent was obtained from all participants.

All patients were assessed for chronic respiratory symptoms, sleep habits, presence of other comorbidities, current and previous medication use, and tobacco exposure, as well as alcohol consumption. A focused clinical examination, that included measurement of anthropometric characteristics, vital signs, and respiratory assessment, was performed. Briefly, parameters such as height, weight, neck circumference, hip and waist circumference, waist/hip circumference ratio, and body mass index (BMI) were measured as part of routine examination at every patient’s visit. 

In addition, lung function was evaluated by pulmonary function testing, via a spirometry device (Chest Co., Tokyo, Japan), and analysis of arterial blood gases, which were collected from the patients’ radial artery, using an ABL3000 auto-analyzer (Radiometer Co., Tokyo, Japan). Cardiac evaluation included a routine 12-lead electrocardiogram the night before polysomnography in all subjects.

The diagnosis of COPD was established in the setting of a post-bronchodilator FEV_1_/FVC ratio of less than 0.7, combined with chronic symptoms, such as dyspnea, cough and/or sputum production, and in association with a history of exposure to risk factors [19]. 

Subjective daytime sleepiness was evaluated via the use of the validated Greek version of the Epworth Sleepiness Scale (ESS) [20], which is a self-administered questionnaire assessing the probability of falling asleep in a variety of daily circumstances (maximum score: 24; score > 10: excessive daytime sleepiness). 

### 2.2. Polysomnography

All included participants underwent an attended 8 h overnight polysomnography (PSG) (from 22:00 to 06:00), which was performed using a laboratory-based instrument (Alice^®^ 4, Philips Respironics, Murrysville, PA, USA). PSG included a standard montage of electroencephalogram, electro-oculogram, electromyogram (submental and bilateral tibial), and electrocardiogram signals. Monitoring of respiratory events was performed using combined oronasal thermistors and thoracic/abdominal strain gauges, while oxyhemoglobin saturation was assessed using a pulse oximeter placed on the index finger. Apneas, hypopneas, and electroencephalogram recordings were manually scored according to standard criteria [21]. Apnea was defined as a ≥90% of reduction in airflow for at least 10 s. Hypopnea was defined as a ≥30% reduction in airflow for at least 10 s in combination with oxyhemoglobin desaturation of at least 3% or an arousal registered by the electroencephalogram. The apnea–hypopnea index (AHI) was regarded as the average number of apneas and hypopneas per hour of PSG-recorded sleep time. OSAS was defined as AHI ≥ 5/h accompanied by related symptoms [21].

### 2.3. Statistical Analysis 

All statistical analyses were performed using Statistical Package for Social Sciences 17.0 (SPSS Inc. Released 2008. SPSS Statistics for Windows, version 17.0, Chicago, IL, USA). Normality of distribution for continuous variables was tested by the Kolmogorov–Smirnov test. Normally distributed quantitative data were expressed as mean ± standard deviation (SD) and those with skewed distribution as median (Interquartile range, IQR: 25th–75th percentile). Comparison of percentages between groups was performed using the chi-square test. Comparisons between means were determined with Student’s *t*-test. In case of skewed distribution, the non-parametric Mann–Whitney test was applied. Statistical significance was defined at 5% (two-tailed *p* < 0.05).

## 3. Results

**Figure 1:**
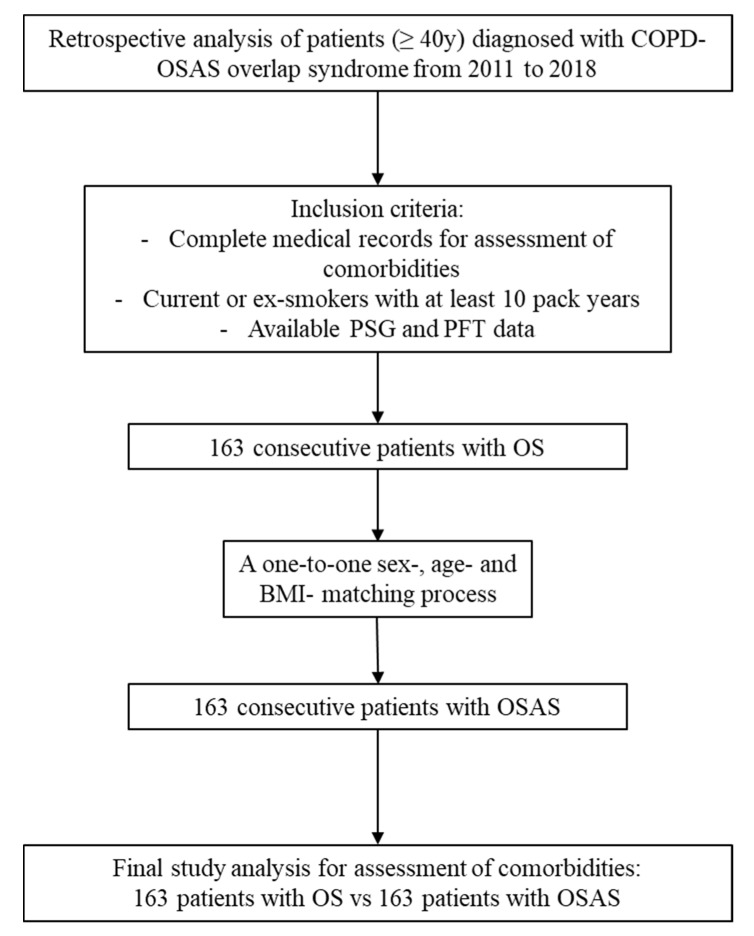
The study flowchart. BMI: body mass index; COPD: chronic obstructive pulmonary disease; OS: overlap syndrome; OSAS: obstructive sleep apnea syndrome; PFTs: pulmonary function testing; PSG: polysomnography.

In total, 163 consecutive OS patients (AHI > 5/h and FEV1/FVC < 0.7) were enrolled from 2011 to 2018 and compared to 163 patients with OSAS (AHI > 5/h and FEV1/FVC > 0.7), after a one-to-one sex, age-, and BMI-matching process during the same study period (Figure 1). The two groups did not differ in terms of: sex (males/females: 139/24 for OSAS; 138/25 for OS, *p* = 0.877), age (59 (51.5–66.5) years for OSAS, 61 (54–68) years for OS, *p* = 0.221), BMI (36.1 (32–39.2) Kg/m^2^ for OSAS, 36.2 (32.1–41) Kg/m^2^ for OS, *p* = 0.496), and smoking history (*p* = 0.412 for current smokers, and *p* = 0.734 for ex-smokers). No differences were observed between the two groups in the anthropometrics’ characteristics except for higher waist circumference in OS patients (122 (110.5–129) for OSAS vs. 126 (114–135) for OS, *p* = 0.018).

Compared to OSAS patients, OS patients demonstrated lower average sleep oxyhemoglobin (*p* = 0.008), higher sleep time with oxygen saturation <90% (*p* = 0.02) during sleep and lower levels of PaO_2_ (*p* < 0.001), and PaCO_2_ (*p* < 0.04) during wakefulness. As expected, impaired lung function was noticed in pulmonary function tests during wakefulness, expressed by Forced Expiratory Volume at 1 s (FEV1) and Forced Vital Capacity (FVC) in OS compared with OSAS (*p* < 0.001 for both). Table 1 displays the anthropometric characteristics, whereas Table 2 and Table 3 report the PSG findings and the results of pulmonary function tests, respectively.

In both OS and OSAS, hypertension was the most prevalent comorbidity (*n* = 96 (58.9%) for OSAS vs. *n* = 101 (62%) for OS, *p* = 0.571), followed by dyslipidemia (*n* = 46 (28.2%) for OSAS vs. *n* = 52 (31.9%) for OS, *p* = 0.469), and diabetes (*n* = 29 (17.8%) for OSAS vs. *n* = 37 (22.7) for OS, *p* = 0.270). OS was characterized by a higher prevalence of total comorbidities (median: 2 (IQR: 1–3) vs. 2 (IQR: 1–2), *p* = 0.033) and particularly CVD, which was the composite of coronary artery disease, heart failure, cerebrovascular, and peripheral artery disease than OSAS (*n* = 22 (13.5%) for OSAS vs. 39 (23.9%) for OS, *p* = 0.016). No differences were observed in the prevalence of other comorbidities between the two groups. Table 4 presents all comorbidities that were found in our study analysis in OSAS and OS patients. 

## 4. Discussion

The present study highlights important findings in the field of overlap syndrome. Firstly, patients with OS demonstrate an increased number of concomitant diseases at their first initial evaluation for sleep-disordered breathing in a sleep laboratory, and higher prevalence of established CVD in OS compared with sex-, age-, and BMI-matched OSAS patients. In addition, components of metabolic syndrome such as hypertension, dyslipidemia, and diabetes similarly occur in both OS and OSAS. Finally, OS compared with OSAS patients demonstrate worse indices of sleep hypoxia and gas exchange in wakefulness. To the best of our knowledge this is the largest study to date on the prevalence of coexisting diseases in the field of overlap syndrome. 

Recent studies have found high occurrence of comorbid diseases in patients with OS, two of them comparing OS and OSA patients [13,14], two others included OS and COPD only individuals [8,22], while one examined and compared all three groups [23]. In the most recent study, OS patients found to have higher prevalence of heart failure than COPD and OSAS subjects (*p* < 0.01; for all), while coronary artery disease was more prevalent in OS than OSAS only individuals (*p* < 0.01) [23]. The findings of this study are in line with our study, which demonstrate the burden of CVD [23]. Nevertheless, the authors matched their patients for age and sex, while in our study BMI was also accounted for in the matching process. In a cross-sectional study [14], that included 38 OS patients and 38 OSAS patients matched for gender, age, and BMI, OS were frequently affected by cardiometabolic diseases, namely CVD, hypertension, diabetes mellitus, and dyslipidemia, while an increased number of several other comorbidities (≥4) was more often observed in OS than OSAS patients (11, vs. 4, respectively). Similarly, Lacedonia et al. [13], in a retrospective study including 989 individuals, of whom 721 had OSAS, 123 OS, and 145 obesity–hypoventilation syndrome, showed that OS are exposed to a higher number of concomitant diseases in contrast to OSAS. Notably, hypertension was the most prevalent disease in OS compared with the other two groups, whereas diagnosis of OS was associated more often with the presence of ≥3 comorbidities compared with OSAS (*p* < 0.001). Steveling et al. [22], in a cross-sectional study that included 177 COPD patients, of whom 33 had coexistent OSA, demonstrated among other observations that diabetes mellitus and arterial hypertension were more frequently present in patients with overlap syndrome than in COPD only patients. Finally, in the landmark study of Marin et al. in the field of overlap syndrome, a higher proportion of patients with OS received antihypertensive treatment and statin therapy and had higher scores in Charlson comorbidity index compared with COPD individuals, indicating frequent presence of multi-morbidity in OS [8]. These findings are in line with our results, underlining that OS is related with a significant burden of cardiometabolic disorders than OSAS or COPD alone. 

In fact, this could be partially explained by the concurrent attribution of COPD and OSAS in the same patient toward the pathogenesis of multiple diseases, particularly CVD [24]. Both diseases are known to predispose to an increased load of systemic inflammation, oxidative stress, endothelial dysfunction and sympathetic over-activity; all are implicated in the pathophysiology of numerous diseases [25,26]. Alone or combined, these mechanisms may increase the risk of systemic hypertension, glucose intolerance, CVD, arrhythmias, neurological and endocrine disease, and cancer in COPD and OSAS patients [11,12]. In a retrospective study [27], that included 146,141 patients with COPD (mean age 67.2 years) compared to one-to-one matched for age and gender randomly selected non-COPD controls, showed that COPD presented more comorbidities than controls, and particularly they were highly affected by hypertension (over 70%), followed by anxiety and depression, diabetes, heart failure, gastroesophageal reflux disease, chronic kidney disease, and osteoporosis. Notably, a significant number of the investigated COPD patients had Charlson Index ≥5 compared to non-COPD individuals (15.57% vs. 5.58, respectively), indicating the increasing burden of accompanied disorders in this population. Multi-morbidity has also been shown to be highly present in patients with OSAS. Several studies strongly relate presence of OSAS, and even increasing severity of the syndrome, with a high occurrence of several comorbidities [11]. In a cross-sectional study [28] in 1929 patients referred to a sleep laboratory for suspected OSAS, of whom 62.1% were diagnosed with OSAS (AHI > 5/h), increasing OSAS severity was significantly associated with higher prevalence of heart attack, coronary artery disease, hypertension, diabetes, and obesity. A large cohort study in the USA [29], enrolling 1,704,905 individuals with a diagnosis of OSAS from a nationwide database and a matched randomly selected sample of controls (*n* = 1,704,417), revealed that OSAS patients had a significantly higher prevalence of important comorbidities-namely type 2 diabetes, cardiovascular disease, hypertension, arrhythmias, and depression, than controls and this finding was more apparent with increasing age.

In the case of OS these patho-physiological phenomena might all simultaneously emerge resulting in the presence and/or development of co-existing diseases [10], as it is shown in our study. Regarding pathogenesis of CVD, both COPD and OSAS are linked with systemic inflammation, oxidative stress, and hyper-coagulopathy, which predispose to endothelial dysfunction and atherosclerosis [1,30]. Moreover, COPD via smoking and OSAS via obesity could further promote development of CVD [1]. All these taken together multiply the risk of CVD in patients with OS. Indeed, in our study established CVD was shown to be more often present in OS than OSAS patients (*p* = 0.016). Despite that there were no differences in terms of prevalence of other diseases between the OS and OSAS groups, common comorbidities, such as hypertension, diabetes, and dyslipidemia, were highly prevalent in the group of patients with OS, corroborating the findings of previous reports. Specifically, both the studies of Lacedonia et al. [13] and Papachatzakis et al. [14], assessing the prevalence of comorbidities in OS, reported that besides occurrence of established CVD, hypertension, diabetes, and dyslipidemia, all components of metabolic syndrome, were the most representative comorbidities in this group of patients, indicating a clear association between OS and cardiometabolic diseases. Interestingly, several other comorbidities, even in a small portion of OS patients, were found also to be present in our study analysis, such as thyroid diseases, depression, gastroesophageal reflux, and connective tissue disease. Perhaps, a future study enrolling more patients with OS could identify stronger or weaker associations between OS and other comorbidities. 

Regarding the breathing function in both wakefulness and during sleep, our findings have shown that both were worse in the group of patients with OS. Specifically, daytime O_2_ and CO_2_ levels were impaired in OS patients, while parameters of oxygen saturation during sleep were also poorer in the same group compared with the OSAS patients. In one of the earlier studies, which was conducted by Chaouat et al. [17], levels of PaO_2_ were lower and PaCO_2_ were higher in awake in OS patients compared with OSAS patients, while mean SpO_2_ levels during sleep were significantly decreased in OS than OSAS individuals. Additionally, other studies have also observed impaired levels of T90% during sleep in OS compared with OSAS [31,32], similar to our results. Moreover, as expected, and by definition, pulmonary function testing was markedly decreased in OS patients due to the presence of COPD. These findings could partially be responsible for the more frequent occurrence of CVD, but also for the coexistence of arterial hypertension and atrial fibrillation in the OS group. An increasing body of evidence acknowledges hypoxia and impaired lung function as important risk factors for the development of CVD, linking also patho-physiologically various pulmonary disorders with poor CV outcomes [26,33]. 

Certainly, the present study is subject to several limitations. Firstly, this is cross-sectional study and hence no specific causality could be extracted regarding the role of OS on the prevalence of comorbidities. Nevertheless, an association between OS and multi-morbidity that is more frequently observed in OS compared with OSAS individuals could be drawn from this study. Secondly, the true prevalence of pulmonary hypertension (PH), assessed by objective evaluation, could not be demonstrated in the present cohort of included patients. We only included information regarding the presence or not of cardiovascular disease, including heart failure as one of the components of CVD, based on the history and/or medical treatment of our patients. Nevertheless, it is now widely known that OS predisposes more often to PH development compared with OSAS [10]. Another limitation is the relatively small number of patients with OS and OSAS assessed for the presence of multiple comorbidities; some of which were even rare to be traced. However, our study attempted to fill the research gap regarding the burden and the different prevalence rates of comorbidities between OS and OSAS patient populations. Therefore, large-scale studies are needed to systematically elucidate the actual burden of multimorbidity in OS and OSAS. Moreover, information regarding prevalence of comorbidities in COPD only patients were lacking from our study. A recent study examined the prevalence of cardiovascular disease in patients with OS compared with COPD (and OSAS), but information was lacking regarding the presence of other comorbidities in these patient groups [23]. Therefore, future evidence is required in order to assess general and specific characteristics, such as PSG events, outcomes, associated diseases, and mortality, between OS and COPD patients [34]. Of interest, a recent report conducted by an ATS panel of experts in this field suggested that studies should evaluate differences between either COPD and OS or OSAS and OS patients, in order to better elucidate the burden of overlap syndrome in various clinical aspects [35]. Finally, we could not demonstrate whether treatment with positive airway pressure (PAP) might have influenced the prevalence of comorbidities in our cohort and whether PAP therapy could eliminate several coexisting diseases, which are shown to be associated with either OSA or COPD, and contribute in reducing the cardiometabolic risk. The latter remains to be investigated in a future prospective study. 

## 5. Conclusions

To conclude, this study shows that OS is accompanied by an increased number of total comorbidities and a higher prevalence of CVD in comparison to sex-, age-, and BMI-matched OSAS patients. Thus, investigation of patients with overlap syndrome for concomitant diseases seems to be highly reasonable at their initial assessment in order for these disorders to be early identified and properly managed. Ultimately, this approach might lead to reduced morbidity and mortality of this complex association between OSAS and COPD. 

## Figures and Tables

**Table 1:** Comparison of anthropometric characteristics between patients with obstructive sleep apnea syndrome (OSAS) and patients with overlap syndrome (OS).

	OSAS Patients *n* = 163	Overlap Syndrome Patients *n* = 163	*p*
Gender (males/females)	139/24	138/25	0.877
Age (years)	59 (51.5–66.5)	61 (54–68)	0.221
BMI (kg/m^2^)	36.1 (32–39.2)	36.2 (32.1–41)	0.496
Neck circumference (cm)	45 (42–48)	45 (42–48)	0.922
Waist circumference (cm)	122 (110.5–129)	126 (114–135)	0.018
Hip circumference (cm)	119 (111–126)	118 (111–128	0.972
WHR	0.86 (0.80–0.96)	0.89 (0.80–0.96)	0.377
Smoking status			
Never smokers (%)	31.3%	25.2%	0.219
Current smokers (%)	63.1%	61.3%	0.631
Ex-smokers (%)	36.9%	38.7%	0.734

Abbreviations: BMI: Body mass index; WHR: Waist to hip ratio.

**Table 2:** Comparison of sleep characteristics between patients with obstructive sleep apnea syndrome (OSAS) and patients with overlap syndrome (OS).

	OSAS Patients *n* = 163	Overlap Syndrome Patients *n* = 163	*p*
Recording time (min)	378 (356–404)	383 (336–402)	0.665
TST (min)	306 (248.5–335.5)	309.8 (253.3–349.6)	0.152
N1 (%)	18.8 (9–34.5)	15.6 (7.8–31.9)	0.308
N2 (%)	59.8 (45.6–72.4)	60.6 (45.4–73.3)	0.646
N3 (%)	5.6 (0–14.6)	6.3 (0.03–12.9)	0.939
REM (%)	9.6 (3.9–14.5)	7.6 (2.4–15)	0.275
AHI (events/h)	35.2 (16.8–62.3)	36.8 (16–61)	0.346
ODI (events/h)	46.3 (26.5–63.1)	45.9 (22.5–69.9)	0.668
Aver SpO_2_ (%)	92 (90–93)	91 (88–93)	0.008
Min SpO_2_ (%)	78 (70–83)	75 (66.3–82)	0.100
T < 90% (min)	12.1 (3.4–35.9)	22.3 (4.8–55.1)	0.020
Arousal index	31.3 (22–45.7)	27.3 (12–45.2)	0.110
Sleep efficiency (%)	80.8 (69.2–86.6)	81.8 (69.2–89.4)	0.300
ESS score	9 (6–14)	9 (6–14)	0.988

Abbreviations: AHI: apnea hypopnea index; Aver SpO_2_: average oxyhemoglobin saturation; ESS: Epworth sleepiness scale; Min SpO_2_: minimum oxyhemoglobin saturation; N1: sleep stage 1; N2: sleep stage 2; N3: sleep stage 3; ODI: oxygen desaturation index, REM: rapid eye movement; TST: total sleep time; T < 90%: time with oxyhemoglobin saturation <90%.

**Table 3:** Respiratory function during wakefulness between patients with obstructive sleep apnea syndrome (OSAS) and patients with overlap syndrome (OS).

	OSAS Patients *n* = 163	Overlap Syndrome Patients *n* = 163	*p*
FEV1 (% predicted)	97.8 (86.8–110)	68.1 (51.4–79.3)	<0.001
FVC (% predicted)	87.9 (77–100)	73.3 (57.9–89)	<0.001
FEV_1_/FVC (%)	85.8 (82.2–90.4)	67.2 (62.3–69.2)	<0.001
Airflow limitation			
Mild (%)	-	24.5%	-
Moderate (%)	-	57.7%	-
Severe (%)	-	17.8%	-
pH	7.42 (7.40–7.44)	7.42 (7.41–7.44)	0.827
pO_2_ (mmHg)	77 (70–83.2)	71.6 (64–79.5)	<0.001
pCO_2_ (mmHg)	41 (39–44)	43 (39–44)	0.040

Abbreviations: FEV1: forced expiratory volume in the first second; FVC: forced vital capacity; pCO_2_: partial carbon dioxide pressure; pO_2_: partial oxygen pressure.

**Table 4:** Prevalence of comorbidities between patients with obstructive sleep apnea syndrome (OSAS) and patients with overlap syndrome (OS).

	OSAS Patients *n* = 163	Overlap Syndrome Patients *n* = 163	*p*
Comorbidities total	2 (1–2)	2 (1–3)	0.033
No comorbidities (*n*, %)	34 (20.9)	24 (14.7)	0.148
Hypertension (*n*, %)	96 (58.9)	101 (62)	0.571
Cardiovascular disease (*n*, %)	22 (13.5)	39 (23.9)	0.016
Dementia (*n*, %)	1 (0.6)	1 (0.6)	1
Cerebrovascular disease (*n*, %)	2 (1.2)	2 (1.2)	1
Diabetes (*n*, %)	29 (17.8)	37 (22.7)	0.270
Dyslipidemia (*n*, %)	46 (28.2)	52 (31.9)	0.469
Atrial fibrillation (*n*, %)	7 (4.3)	14 (8.6)	0.114
Cancer (*n*, %)	0 (0)	3 (1.8)	0.082
Depression (*n*, %)	10 (6.1)	7 (4.3)	0.455
Gastroesophageal reflux (*n*, %)	7 (4.3)	6 (3.7)	0.777
Connective tissue disease (*n*, %)	3 (1.8)	6 (3.7)	0.311
Chronic kidney disease (*n*, %)	1 (0.6)	1 (0.6)	1
Liver disease (*n*, %)	1 (0.6)	0 (0)	0.317
Osteoporosis (*n*, %)	3 (1.8)	2 (1.2)	0.652
Hyperuricemia (*n*, %)	6 (3.7)	4 (2.5)	0.521
Thyroid gland disease (*n*, %)	13 (8)	18 (11)	0.345
Parkinson disease (*n*, %)	3 (1.8)	0 (0)	0.082

## Data Availability

All data have been presented in this manuscript.
